# Binomial nomenclature for virus species: a consultation

**DOI:** 10.1007/s00705-019-04477-6

**Published:** 2019-12-03

**Authors:** Stuart G. Siddell, Peter J. Walker, Elliot J. Lefkowitz, Arcady R. Mushegian, Bas E. Dutilh, Balázs Harrach, Robert L. Harrison, Sandra Junglen, Nick J. Knowles, Andrew M. Kropinski, Mart Krupovic, Jens H. Kuhn, Max L. Nibert, Luisa Rubino, Sead Sabanadzovic, Peter Simmonds, Arvind Varsani, Francisco Murilo Zerbini, Andrew J. Davison

**Affiliations:** 1 School of Cellular and Molecular Medicine, Faculty of Life Sciences, University of Bristol, University Walk, Bristol, BS8 1TD UK; 2 School of Biological Sciences, The University of Queensland, Saint Lucia, QLD 4072 Australia; 3 Department of Microbiology, University of Alabama at Birmingham (UAB), BBRB 276, 845 19th ST South, Birmingham, AL 35294-2170 USA; 4 Division of Molecular and Cellular Biosciences, National Science Foundation, 2415 Eisenhower Avenue, Alexandria, VA 22314 USA; 5 Theoretical Biology and Bioinformatics, Department of Biology, Utrecht University, Padualaan 8, Room N-604, 3584 CH Utrecht, The Netherlands; 6 Centre for Molecular and Biomolecular Informatics, Radboud University Medical Center (Radboudumc), Geert Grooteplein 26, 6525 GA Nijmegen, The Netherlands; 7 Institute for Veterinary Medical Research, Centre for Agricultural Research, Hungária krt. 21, Budapest, 1143 Hungary; 8 Invasive Insect Biocontrol and Behavior Laboratory, USDA-ARS, 10300 Baltimore Avenue, Bldg 007 BARC-West, Beltsville, MD 20705 USA; 9 Institute of Virology, Charité-Universitätsmedizin, Corporate Member of Free University Berlin, Humboldt-University Berlin, Berlin, Germany; 10 Berlin Institute of Health, Berlin, Germany; 11 The Pirbright Institute, Ash Road, Pirbright, Surrey, GU24 0NF UK; 12 Department of Food Science, University of Guelph, Guelph, ON N1G 2W1 Canada; 13 Department of Pathobiology, University of Guelph, Guelph, ON N1G 2W1 Canada; 14 Department of Microbiology, Institut Pasteur, 25 rue du Dr Roux, 75015 Paris, France; 15 National Institutes of Health, National Institute of Allergy and Infectious Diseases, Division of Clinical Research, Integrated Research Facility at Fort Detrick (IRF-Frederick), B-8200 Research Plaza, Fort Detrick, Frederick, MD 21702 USA; 16 Department of Microbiology, Blavatnik Institute, Harvard Medical School, 77 Ave Louis Pasteur, Boston, MA 02115 USA; 17 Istituto per la Protezione Sostenibile delle Piante, CNR, Sede Secondaria di Bari, Via Amendola 165/A, 70126 Bari, Italy; 18 Department of Biochemistry, Molecular Biology, Entomology and Plant Pathology, Mississippi State University, 100 Old Hwy 12 Mail Stop 9775, Mississippi State, MS 39762 USA; 19 Nuffield Department of Experimental Medicine, University of Oxford, Peter Medawar Building, South Parks Road, Oxford, OX1 3PS UK; 20 The Biodesign Center for Fundamental and Applied Microbiomics, School of Life Sciences, Center for Evolution and Medicine, Arizona State University, P.O. Box 874701, Tempe, AZ 85287-4701 USA; 21 Departamento de Fitopatologia/BIOAGRO, Universidade Federal de Viçosa, Viçosa, MG 36570-900 Brazil; 22 MRC-University of Glasgow Centre for Virus Research, Sir Michael Stoker Building, 464 Bearsden Road, Glasgow, G61 1QH UK

**Keywords:** Virus taxonomy, Species nomenclature, Nomenclature, Binomial species names, International committee on taxonomy of viruses (ICTV)

## Abstract

The Executive Committee of the International Committee on Taxonomy of Viruses (ICTV) recognizes the need for a standardized nomenclature for virus species. This article sets out the case for establishing a binomial nomenclature and presents the advantages and disadvantages of different naming formats. The Executive Committee understands that adopting a binomial system would have major practical consequences, and invites comments from the virology community before making any decisions to change the existing nomenclature. The Executive Committee will take account of these comments in deciding whether to approve a standardized binomial system at its next meeting in October 2020. Note that this system would relate only to the formal names of virus species and not to the names of viruses.

## Introduction

The International Committee on Taxonomy of Viruses (ICTV; http://www.ictv.global) was founded in 1966 as the International Committee on Nomenclature of Viruses. The ICTV has the following objectives.

1. To develop an internationally agreed taxonomy for viruses.
2. To establish internationally agreed names for virus taxa.
3. To communicate the decisions reached concerning the classification and nomenclature of viruses to virologists by holding meetings and publishing reports.
4. To maintain an official index of agreed names of virus taxa.

Among the rules adopted at an early stage by the International Committee on Nomenclature of Viruses were several that seemed to be aimed at realizing the first two objectives in the form of a standardized nomenclature. The following rules were included:

1. Nomenclature shall be international.
2. Nomenclature shall be universally applied to all viruses.
3. An effort will be made towards a Latinized binomial nomenclature.
4. Existing Latinized names shall be retained whenever feasible.

However, subsequent attempts at establishing a standardized nomenclature, whether Latinized or non-Latinized, whether binomial or otherwise, have failed. As a result, there exists no standardized format for the names of virus species. In contrast, the nomenclature of higher taxa is standardized, consisting of single words ending with rank-specific suffixes. The International Code of Virus Classification and Nomenclature specifies the following requirements.

1. A species name shall consist of as few words as practicable but be distinct from the names of other taxa. Species names shall not consist only of a host name and the word “virus”.
2. Species names are printed in italics and have the first letter of the first word capitalized. Other words are not capitalized unless they are proper nouns or parts of proper nouns.
3. A species name must provide an appropriately unambiguous identification of the species.

This state of affairs has resulted in decades of debate on the desirability and format of a standardized naming system for virus species [1–5]. The ICTV Executive Committee has considered this situation [6] and recognizes that the need for such a system has become overwhelming, not only because of the huge number of viruses that are inferred to exist from environmental DNA sequencing studies but also because of the benefits of distinguishing more clearly between species and virus names.

This article aims to set out the case for establishing a binomial nomenclature for virus species (and, by extension, satellite and viroid species), to explain the advantages and disadvantages of alternative naming formats, and to invite comments from the virology community before making any decisions on changes to existing nomenclature. Before embarking on this exercise, it is essential to emphasize the distinction between a virus and a virus species. A virus is a physical entity that occurs naturally, infects a host, and may cause disease. A virus species is an abstract taxonomic category to which a virus is assigned. Thus, a virus is not a species but is assigned taxonomically to a species. During its history, the ICTV mandate has been redefined from naming viruses to naming virus species, and, as a result, this article deals exclusively with the latter.

## Binomial nomenclature

A binomial nomenclature is a formal way of naming species of living things by giving each a name composed of two parts. In all areas of biology except virology, the first part of the name consists of the name of the genus to which the species belongs, and the second part (the specific or species epithet) identifies the species within that genus. For example, humans belong to the genus *Homo* and within this genus to the species *Homo sapiens*. Moreover, the two parts are Latinized, taking Latin grammatical forms, although they can be based on words from other languages.

In most branches of biology, the assignment of a species name is determined by the priority of a valid publication describing a specimen and is associated with a physical type specimen. For these reasons, species names are often followed (particularly on first use in a publication) by an authority (the name of the author of the description) and sometimes by a date. However, priority has never been incorporated into descriptions of virus species, as both taxonomy and nomenclature are adopted simultaneously by decisions of the ICTV. Therefore, this article discusses only the format of species names; there is no intention to adopt the concept of authorities in these names or to require physical type specimens.

## Current nomenclature

Virus species names are currently rarely Latinized and take a variety of forms, examples of which are listed in Table 1. Some names incorporate the name of the genus into which the virus species is classified, but this is not applied consistently. Some species names look like genus names but are not (e.g., *Lausannevirus*). Some species names are single words, some are binomial, and some are multinomial. Some include single or multiple letters or numbers at various positions or have Latinized elements (most often as a name or a part of a name of a host taxon) and non-Latinized elements. Moreover, some have identical suffixes (‘…*virus*’) in more than one component (e.g., *Senegalvirus marseillevirus*).

**Table 1:** Forms of species name

Species name	Form
*Escherichia virus T4*	Host genus + virus + phage name
*Suid alphaherpesvirus 1*	Host family (part) + subfamily + number
*Mammalian 1 bornavirus*	Host group + number + genus
*Alfalfa mosaic virus*	Host common name + symptom + virus
*Alphacoronavirus 1*	Genus + number
*Cardiovirus A*	Genus + letter
*Lambdaarterivirus afriporav*	Genus + acronym
*Cafeteria roenbergensis virus*	Host species + virus
*Potato virus X*	Host common name + virus + letter (not in series)
*Rhizosolenia setigera RNA virus 01*	Host species + genome + virus + number
*Tomato spotted wilt tospovirus*	Host common name + symptom + genus
*Human mastadenovirus C*	Host common name + genus + letter
*Autographa californica multiple nucleopolyhedrovirus*	Host species + virion feature/defunct genus
*Drosophila X virus*	Host genus + letter (not in series) + virus
*Lassa mammarenavirus*	Place + genus
*Senegalvirus marseillevirus*	Place-virus + genus
*Sapporo virus*	Place + virus
*Lausannevirus*	Place-virus
*Rosellinia necatrix quadrivirus 1*	Host species + genus + number
*Colorado tick fever virus*	Disease + virus
*Tomato yellow leaf curl Indonesia virus*	Disease + place + virus

## Adopting binomial nomenclature

### Genus name

In the context of viruses, genus names are already required by the International Code of Virus Classification and Nomenclature to be single words ending in ‘…*virus*’ and therefore could be used without change. For example, *Bean golden yellow mosaic virus* (type species of genus *Begomovirus*, family *Geminiviridae*) could be renamed *Begomovirus +* epithet.

### Specific epithet

The epithet may take a variety of forms, as described below:

#### Latin or Latinized epithet

The epithet would take one of the two following forms.

1. Genuine Latin words. For example, the epithet could be derived from the scientific name of a host organism (e.g., *Cripavirus rhopalosiphi* for a cripavirus that is associated with aphids of the genus *Rhopalosiphum*) or Latin forms of geographical names (e.g., *Begomovirus novodelhiense* or *Begomovirus newdelhiense* for a begomovirus that has links to New Delhi).
2. Latinized words constructed from any root or a portmanteau to create a pseudo-Latin word with an appropriate ending, in the way that many genus names are already constructed (e.g., *Begomovirus tylecundus* from tomato yellow leaf curl New Delhi).

The distinction between Latin and Latinized words is not absolute, but an insistence that the epithet must always be recognizable as a genuine Latin word (i.e., taking form 1) is likely to be too difficult to apply. Notably, no other biological taxonomy insists on epithets having to be in this more restricted form.

Advantages:

1. A Latinized system would be consistent with all other biological taxonomies. Moreover, biologists are used to applying Latinized binomials to taxa.
2. A Latin or Latinized system would make it immediately obvious to all that a name is that of a species. This system would bring a degree of consistency that would be understood and appreciated by specialists (editors, authors, data curators, etc.) and non-specialists alike. Virus names would then be seen as clearly distinct and could exist in any language and in any form (translated, transliterated, original, etc.). In contrast, species names could be represented in the same form in every language.
3. Latin is a historic language with a minimal character set that does not require diacritics and will not change in its syntax. As a result, it is universal, stable, and uses characters that can be typed directly from any keyboard that uses a Roman or Latin script.
4. Because the first word of the binomial name of a virus species is a genus name, it always ends in ‘…*virus*’, and it can be treated as a neuter noun, thus simplifying the task of providing the appropriate ending to the epithet.
5. In case the taxonomy has to be revised and a species has to be moved into a new genus, it would usually be possible to retain the epithet and thereby provide some continuity. Such changes would be relatively easy to track, and biologists have become used to tracking changes in prokaryote and eukaryote taxa. Moreover, during the creation of epithets, it may often be possible to incorporate elements of the existing species name, providing a memorable link between the existing and new species names (as in the examples above).

Disadvantages:

1. Any standard naming system must be easy to apply and use. When Latinized binomials were first adopted in biology, Latin was the international language of science. Indeed, for many years it was necessary to publish formal descriptions of new species (e.g., of plants and mammals) in Latin. Today, few people understand Latin well enough to create species names in the correct form without some basic introduction.
2. It may be difficult to devise epithet names for large numbers of species. This may be regarded as particularly problematic for viruses identified metagenomically. For example, these may entirely lack the phenotypic information that assists classification elsewhere in biology (e.g., host, morphology, and disease associations). The practicalities of devising Latinized epithets in substantial numbers have been examined recently [7].
3. Some would argue that virus species names should be recognizably different in style from those used elsewhere in biology, to affirm the point of view that viruses are not living organisms or that species of viruses may have a different status from those of cellular organisms.

#### Alphanumeric characters in a logical series

The epithet would consist of numbers or letters (e.g., *Begomovirus 127* or *Begomovirus DF*). Such codes are already used in, for example, the families *Papillomaviridae* and *Picornaviridae* (e.g., *Alphapapillomavirus 1* and *Enterovirus A*).

Advantages:

1. Because some species names already have this format, they would not need to change. Also, the idea is familiar.
2. This system provides an easy and infinitely expandable way of naming species and may be relevant to creating large numbers of species names following large-scale environmental sequencing studies.

Disadvantages:

1. The names may not be memorable, even in the short term (e.g., when reading an article or listening to a presentation).
2. There could be difficulties if the taxonomy has to be revised and a species is moved to a new genus. For example, if the (hypothetical) species *Alphamegavirus 5* in a list of ten consecutively numbered species (*1*–*10*) in the genus *Alphamegavirus* were to be moved into the genus *Gammamegavirus* with a list of 20 consecutively numbered species (*1*–*20*), all available renaming options may lead to confusion. Would the name *Alphamegavirus 5* never be filled after the revision, thereby causing discontinuity? Would the name *Alphamegavirus 5* eventually be filled by a novel virus different from the virus that was moved, thereby confusing the literature about the identity of this species name? How would the changes be tracked when the original species name *Alphamegavirus 5* becomes *Gammamegavirus 21* after the revision?
3. Using a letter code instead of a number may pose extra challenges if there were large numbers of species in a genus.
4. In some genera, there would be a risk of confusing species with genotypes or serotypes, which are often distinguished by numbers or letters (e.g., in the family *Picornaviridae*, it is common practice to use this convention for serotypes, such as coxsackievirus B1).

#### Freeform text

Any word would be used as the epithet, free from the constraints of Latinizing. Indeed, the epithet would not need to look like a word but could be any set of characters (e.g., *Begomovirus tylcND1*). The use of freeform text would not exclude the use of the other formats.

Advantages:

1. The system is simple and flexible. Single words related to geographical origin, host, or symptoms (for example) could be used (e.g., *Flavivirus dengue* or *Bymovirus oatmosaic*).
2. In many cases, the binomial form could resemble directly the existing species name (e.g., *Measles morbillivirus* could become *Morbillivirus measles* and *Chikungunya virus* could become *Alphavirus chikungunya*).
3. In particular, it would greatly simplify the derivation of species names for bacterial viruses, where, for example, *Staphylococcus virus SEP1*, in the genus *Sepunavirus*, could become *Sepunavirus SEP1*.

Disadvantages:

1. It may be difficult to devise epithet names for large numbers of species (see *Latin or Latinized epithet*, disadvantage 2).
2. Many names may appear to be uncomfortable hybrids of a pseudo-Latin genus name and something altogether different.
3. The system would appear strange to scientists familiar with the Linnaean system used for most biological species.
4. Many names might be unpronounceable.

## Recommendation

The Executive Committee recommends the adoption of a standardized binomial system for naming virus species in a consistent and universally applied manner. In this system, the species name would consist of two (and only two) words separated by a single space. In this context, a word is defined as a written or printed character or combination of characters. Depending upon the form of the epithet that is chosen, both words would either consist only of the 26 letters of the standard Latin-script English alphabet without diacritical marks or would also allow epithets containing or consisting of Arabic numerical digits.

The first word would be the genus name, which the International Code of Virus Classification and Nomenclature requires to be a single word ending in ‘…*virus*’. The Code also requires all new species to be assigned to genera but, for historical reasons, a number of existing species were not so assigned and it would be necessary to correct this anomaly. Subgenus names also end in ‘…*virus*’ but they would not form the first word of a species name.

The second word, the specific epithet, would be a single word or set of characters that is unique within the genus. Depending upon the form of the epithet that is chosen, the current rules regarding capitalization in the epithet may need to be revised.

At this time, the form of the epithet remains undecided. However, the Executive Committee is considering whether to adopt one of the three alternatives described above:

1. Genus + Latin or Latinized epithet
2. Genus + alphanumeric epithet
3. Genus + freeform epithet

In addition to providing internal consistency in virus taxonomy, any of these formats would bring species names much more into line with those used in all other branches of biology. None would involve changing the names of higher taxa from genus upwards, and, as emphasized above, none would involve changing the names of viruses.

## Implementation

The Executive Committee understands that adopting a binomial system would have major practical consequences, not least in changing the names of most of the 5560 currently classified species. However, it believes that these consequences will be outweighed by the benefits of having a sustainable, standardized system that can function effectively into the future. Importantly, any changes will be accompanied by an implementation period that will allow adequate time for ICTV Study Groups, supported by the Executive Committee, to agree upon new species names that can then be ratified by the ICTV. Different Study Groups would face different hurdles, and the implementation period may vary accordingly. Also, all historical names of species and higher taxa will remain accessible via the ICTV online databases (https://ictv.global).

## Feedback

The Executive Committee invites all virologists to provide comments and opinions on the recommendation above. To facilitate this invitation, a public forum has been established at https://ictv.global/discussion/binomial. [You will be required to register on the site to post comments.] Respondents are also encouraged to read taxonomic proposal 2018.001G.Ud.v2.binomial_species [8] (or the most recent version), which opts for a Latinized binomial system and has already been discussed (but not yet decided upon) by the Executive Committee. This document and others listed in the References can be downloaded from https://ictv.global/files/binomial. At its next meeting in October 2020, the Executive Committee will decide whether to approve 2018.001G.Ud.v2.binomial_species (or the most recent version), taking into account feedback provided by 30 June 2020. Comments may also be emailed directly to the ICTV President (binomials@btinternet.com). These will not enter the public domain but will be shared among Executive Committee members, in an anonymous form if this is requested.
