# Correction to: Binomial nomenclature for virus species: a consultation

**DOI:** 10.1007/s00705-020-04555-0

**Published:** 2020-02-17

**Authors:** Stuart G. Siddell, Peter J. Walker, Elliot J. Lefkowitz, Arcady R. Mushegian, Bas E. Dutilh, Balázs Harrach, Robert L. Harrison, Sandra Junglen, Nick J. Knowles, Andrew M. Kropinski, Mart Krupovic, Jens H. Kuhn, Max L. Nibert, Luisa Rubino, Sead Sabanadzovic, Peter Simmonds, Arvind Varsani, Francisco Murilo Zerbini, Andrew J. Davison

**Affiliations:** 1 School of Cellular and Molecular Medicine, Faculty of Life Sciences, University of Bristol, University Walk, Bristol, BS8 1TD UK; 2 School of Biological Sciences, The University of Queensland, Saint Lucia, QLD 4072 Australia; 3 Department of Microbiology, University of Alabama at Birmingham (UAB), BBRB 276, 845 19th ST South, Birmingham, AL 35294-2170 USA; 4 Division of Molecular and Cellular Biosciences, National Science Foundation, 2415 Eisenhower Avenue, Alexandria, VA 22314 USA; 5 Theoretical Biology and Bioinformatics, Department of Biology, Utrecht University, Padualaan 8, Room N-604, 3584 CH Utrecht, The Netherlands; 6 Centre for Molecular and Biomolecular Informatics, Radboud University Medical Center (Radboudumc), Geert Grooteplein 26, 6525 GA Nijmegen, The Netherlands; 7 Institute for Veterinary Medical Research, Centre for Agricultural Research, Hungária krt. 21, Budapest, 1143 Hungary; 8 Invasive Insect Biocontrol and Behavior Laboratory, USDA-ARS, 10300 Baltimore Avenue, Bldg 007 BARC-West, Beltsville, MD 20705 USA; 9 Institute of Virology, Charité-Universitätsmedizin, Corporate Member of Free University Berlin, Humboldt-University Berlin, Berlin, Germany; 10 Berlin Institute of Health, Berlin, Germany; 11 The Pirbright Institute, Ash Road, Pirbright, Surrey, GU24 0NF UK; 12 Department of Food Science, University of Guelph, Guelph, ON N1G 2W1 Canada; 13 Department of Pathobiology, University of Guelph, Guelph, ON N1G 2W1 Canada; 14 Department of Microbiology, Institut Pasteur, 25 rue du Dr Roux, 75015 Paris, France; 15 National Institutes of Health, National Institute of Allergy and Infectious Diseases, Division of Clinical Research, Integrated Research Facility at Fort Detrick (IRF-Frederick), B-8200 Research Plaza, Fort Detrick, Frederick, MD 21702 USA; 16 Department of Microbiology, Blavatnik Institute, Harvard Medical School, 77 Ave Louis Pasteur, Boston, MA 02115 USA; 17 Istituto per la Protezione Sostenibile delle Piante, CNR, Sede Secondaria di Bari, Via Amendola 165/A, 70126 Bari, Italy; 18 Department of Biochemistry, Molecular Biology, Entomology and Plant Pathology, Mississippi State University, 100 Old Hwy 12 Mail Stop 9775, Mississippi State, MS 39762 USA; 19 Nuffield Department of Experimental Medicine, University of Oxford, Peter Medawar Building, South Parks Road, Oxford, OX1 3PS UK; 20 The Biodesign Center for Fundamental and Applied Microbiomics, School of Life Sciences, Center for Evolution and Medicine, Arizona State University, P.O. Box 874701, Tempe, AZ 85287-4701 USA; 21 Departamento de Fitopatologia/BIOAGRO, Universidade Federal de Viçosa, Viçosa, MG 36570-900 Brazil; 22 MRC-University of Glasgow Centre for Virus Research, Sir Michael Stoker Building, 464 Bearsden Road, Glasgow, G61 1QH UK

## Correction to: Archives of Virology (2020) 165:519–525 10.1007/s00705-019-04477-6

The article Binomial nomenclature for virus species: a consultation, written by Stuart G. Siddell, Peter J. Walker, Elliot J. Lefkowitz, Arcady R. Mushegian, Bas E. Dutilh, Balázs Harrach, Robert L. Harrison, Sandra Junglen, Nick J. Knowles, Andrew M. Kropinski, Mart Krupovic, Jens H. Kuhn, Max L. Nibert, Luisa Rubino, Sead Sabanadzovic, Peter Simmonds, Arvind Varsani, Francisco Murilo Zerbini, Andrew J. Davison, was originally published Online First without Open Access. After publication in volume 165, issue 2, page 519-525 the author decided to opt for Open Choice and to make the article an Open Access publication. Therefore, the copyright of the article has been changed to © The Author(s) 2020 and the article is forthwith distributed under the terms of the Creative Commons Attribution 4.0 International License (http://creativecommons.org/licenses/by/4.0/), which permits use, duplication, adaptation, distribution and reproduction in any medium or format, as long as you give appropriate credit to the original author(s) and the source, provide a link to the Creative Commons license, and indicate if changes were made.

